# Post-traumatic cavernous hemangioma of the frontal cranial vault

**DOI:** 10.11604/pamj.2014.17.90.3904

**Published:** 2014-02-04

**Authors:** Ali Akhaddar, Jawad Laaguili

**Affiliations:** 1 Department of Neurosurgery, Avicenne Military Hospital, Marrakech, Morocco; 2 Department of Neurosurgery, Mohammed V Military Teaching Hospital, Rabat, Morocco; 3 University of Mohammed V Souissi, Rabat, Morocco

**Keywords:** Traumatic, cavernous hemangioma, cranial vault

## Image in medicine

A 37-year-old previously healthy man presented with a localized headache and a progressive enlarging mass in the left frontal area for more than 8 months. He also reported a history of head injury in the same area sustained two years before. On physical examination, the mass (about 28 mm in diameter) was hard to pressure with freely mobile skin above the lesion in the left frontal sus-orbital region. Ophthalmologic and neurologic examinations were normal. Cranial computed tomography scan showed an osteolytic intradiploic lesion with poorly defined margins (A and B). Magnetic resonance imaging showed hyperintense lesion on T2-weighted image and hypointense on T1-weighted image with gadolinium enhancement (C). The lesion was extradural with slight brain mass effect. Surgery consisted of total resection of the bony bluish lesion (D) and cranioplasty. The postoperative course was uneventful. Histological study revealed a cavernous hemangioma of the diploe. Bone hemangioma is a vascular hemartoma: a benign tumor arising from the intrinsic vasculature of the bone, commonly seen in long bones and vertebrae. Skull cavernous hemangiomas are rare tumors for which the origin is not yet clear. We suspected that in our case head injury may have been the cause of cavernous hemangioma in the cranial vault.

**Figure 1:**
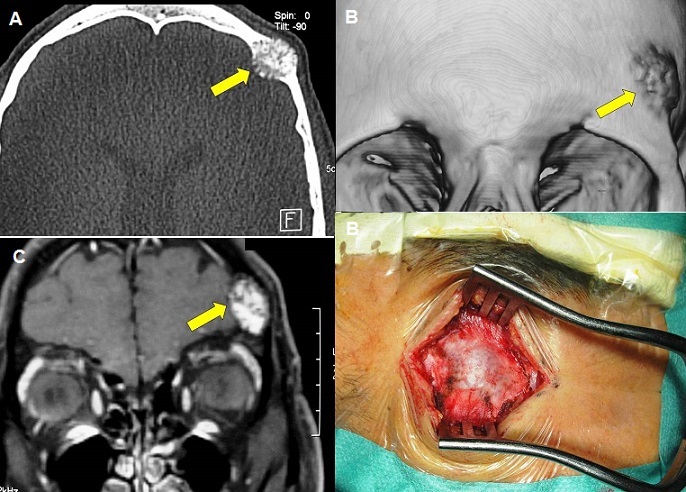
Cranial CT-scan on axial view (bony window) (A) and 3-D bony reconstruction (B) showing an osteolytic intradiploic lesion with poorly defined margins in the left frontal sus-orbital region. Coronal T1-weighted MRI after gadolinium administration showing the hyperintense extradural lesion with slight brain mass effect (C). Operative view of the bony bluish lesion before resection (D)

